# Efficacy and safety comparison between liraglutide as add-on therapy to insulin and insulin dose-increase in Chinese subjects with poorly controlled type 2 diabetes and abdominal obesity

**DOI:** 10.1186/1475-2840-11-142

**Published:** 2012-11-15

**Authors:** Chun-jun Li, Jing Li, Qiu-mei Zhang, Lin Lv, Rui Chen, Chun-feng Lv, Pei Yu, De-min Yu

**Affiliations:** 1 2011 Collaborative Innovation Center of Tianjin for Medical Epigenetics, Key Laboratory of Hormone and Development (Ministry of Health), Metabolic Disease Hospital & Tianjin Institute of Endocrinology, Tianjin Medical University, Tianjin, 300070, China

**Keywords:** Liraglutide, Abdominal obesity, Insulin therapy, Weight reduction

## Abstract

**Objective:**

To assess the efficacy and safety of adding liraglutide to established insulin therapy in poorly controlled Chinese subjects with type 2 diabetes and abdominal obesity compared with increasing insulin dose.

**Methods:**

A 12-week, randomized, parallel-group study was carried out. A total of 84 patients completed the trial who had been randomly assigned to either the liraglutide-added group or the insulin-increasing group while continuing current insulin based treatment. Insulin dose was reduced by 0-30% upon the initiation of liraglutide. Insulin doses were subsequently adjusted to optimized glycemic control. Glycosylated hemoglobin (HbA_1c_) values, blood glucose, total daily insulin dose, body weight, waist circumference, and the number of hypoglycemic events and adverse events were evaluated.

**Results:**

At the end of study, the mean reduction in HbA_1c_ between the liraglutide-added group and the insulin-increasing group was not significantly different (1.9% vs. 1.77%, p>0.05). However, the percentage of subjects reaching the composite endpoint of HbA1c ≤ 7.0% with no weight gain and no hypoglycemia, was significantly higher in the liraglutide-added group than in the insulin-increasing group (67% vs. 19%, p<0.001). Add-on liraglutide treatment significantly reduced mean body weight (5.62 kg, p<0.01), waist circumference (5.70 cm, p<0.01), body mass index (BMI) (1.93 kg/m^2^, p<0.01) and daily total insulin dose (dropped by 66%) during 12-week treatment period, while all of these significantly increased with insulin increasing treatment. Add-on liraglutide treated patients had lower rate of hypoglycemic events and greater insulin and oral antidiabetic drugs discontinuation. Gastrointestinal disorders were the most common adverse events in the liraglutide added treatment, but were transient.

**Conclusions:**

Addition of liraglutide to abdominally obese, insulin-treated patients led to improvement in glycemic control similar to that achieved by increasing insulin dosage, but with a lower daily dose of insulin and fewer hypoglycemic events. Adding liraglutide to insulin also induced a significant reduction in body weight and waist circumference. Liraglutide combined with insulin may be the best treatment option for poorly controlled type 2 diabetes and abdominal obesity.

## Introduction

The prevalence of obesity and diabetes has rapidly increased worldwide Western and Asian countries [1,2]. The association between type 2 diabetes (T2D) and overweight/obesity is indisputable. In China, the prevalence of overweight has increased nearly 50% in a 10-y period [3]. Obesity, especially visceral fat adiposity, also increases the risk of T2D, hypertension and atherosclerosis, suggesting that obese patients with T2D are at high risk for cardiovascular disease (CVD) [4]. In addition, Chinese have more risk factors for CVD because they have relatively more visceral fat at the same body mass index (BMI) than Europeans do [5].

Obese patients with T2D ultimately require insulin therapy to achieve glycemic control over time. However, insulin treatment is commonly associated with hypoglycemia and weight gain. Moreover, 70% of insulin-induced weight gain is fat mass, which in turn worsens insulin resistance. This then leads to further increases in insulin requirement and weight gain. A meta-analysis of waist circumference as predictor of cardiovascular events has shown that a waist circumference increase of 1 cm may be associated with a 2% risk increase in cardiovascular events [6]. In this sense, it is necessary to develop effective and efficient therapeutic strategy for T2D and abdominal obesity as well as decreasing cardiovascular risk.

Glucagon-like peptide-1 (GLP-1) receptor agonists are the newest class of T2D therapy currently available, which improve hyperglycemia through increasing insulin secretion and reducing glucagon secretion [7,8], slowing gastric emptying, delaying carbohydrate absorption, and increasing satiety, leading to reduced caloric intake [9]. In Liraglutide Effect and Action in Diabetes (LEAD) 1–6 trials, substantial and sustained improvement in HbA1c, fasting plasma glucose and postprandial glucose have been observed with liraglutide treatment [10-15]. Several studies have shown that liraglutide treatment results in greater reduction in fat mass than in the lean body mass. Furthermore, visceral fat mass decreases more than abdominal subcutaneous fat mass [11,12,16]. Therefore liraglutide might be a promising new agent for the treatment of T2D and abdominal obesity linked to high risk of CVD.

The combination of exenatide with insulin has been shown to have an insulin-sparing effect in insulin-treated patients with T2D as well as a weight loss benefit in several small uncontrolled studies, furthermore, these studies reported a low incidence of severe hypoglycemia [17]. However, only one small retrospective study had shown that adding liraglutide to insulin therapy resulted in a significant improvement in glycemic control, reduction in insulin requirement, and reduction of body weight without significant hypoglycemia [18]. Therefore, the present study compared the efficacy and safety of adding liraglutide versus increasing insulin dose strategy in insulin-treated poorly controlled T2D and abdominal obesity.

## Materials and methods

### Subjects

This study was undertaken in the out-patient setting of the Metabolic Disease Hospital of Tianjin Medical University between October 2011 and May 2012. Patients eligible for the study met the following criteria: diagnosis of T2D defined by America Diabetes Association in 2003; HbA1c 7.5~11%; BMI ≥ 25 kg/m^2^, waist circumference (male ≥ 90 cm and female ≥ 85 cm). The eligible patients also had to have received insulin injections for at least 3 months at a dose of at least 10 U/day. Through reviewing previous medical history, we excluded those with type 1 diabetes, gestational diabetes or diabetes with identification secondary causes, significant renal impairment (estimated creatinine clearance <50 ml/min) or elevated (≥ 100) alanine or aspartate aminotransferase (ALT or AST) or congestive heart failure (NYHA Class III or IV). Patients who were taking medications, aside from antidiabetic medications, known to affect glycemic or weight control, such as glucocorticoids or orlistat, were also excluded.

### Study design

This was a parallel-group, open-label, randomized clinical trial over a 12-week observation period. All patients provided written informed consent and confirmed their willingness to perform glucose self-monitoring. This study design was approved by the local ethics committee review board and was conducted using Good Clinical Practice in accordance with the Declaration of Helsinki. The eligible patients were randomized to either the liraglutide-added group or the insulin-increasing group on the basis of computer-generated random numbers, by a person not involved in recruitment of patients. The withdrawal rate was assumed to be 10%. The glycemic control target was defined as fasting blood glucose (FBG) ≤6.1 mmol/L and 2 hour postprandial blood glucose (P2BG) ≤8 mmol/L. In the liraglutide-added group, liraglutide was initiated at a dose of 0.6 mg injected subcutaneously once per day and increased to 1.2 mg/day after 1 week. Insulin doses were reduced 0~30% upon initiation of liraglutide based upon the prescribing diabetologists’ judgement. In the insulin-increasing group, insulin doses were increased to reach the glycemic targets. During the study, patients continued their usual diet and exercise regimens as well as any concomitant glucose-lowering medications. The dialectologists optimized liraglutide, insulin dose and antidiabetic agents regimen according to their judgment based on each patient’s glucose and not according to a specific protocol. The dialectologists can first decide to discontinue oral insulin secretagogues in the events of hypoglycemia or hypoglycemic symptoms occurred in the daytime; if hypoglycemia occurred in the nighttime, the investigators can consider decreasing insulin dose or discontinuing insulin treatment according to their clinical judgements. So this study treatment reflects the real-world practice. Patients were seen in follow up at baseline, 2, 4, 8 and 12 weeks.

### Clinical measurements

Clinical parameters evaluated at baseline and at 3 months included HbA1c, total daily insulin dose, total-triglyceride (TG), total-cholesterol, LDL-cholesterol and HDL-cholesterol. Body weight, waist circumference, FBG and P2BG were measured at every clinic visit (baseline, at 2, 4, 8 and 12 weeks). Hypoglycemic episodes and adverse events (AEs) were recorded throughout the study. All patients were taught how to recognize the signs and symptoms of hypoglycemia and instructed to obtain a blood glucose reading whenever symptoms of hypoglycemia occurred. Hypoglycemia was determined by the number of plasma glucose readings that were below 3.9 mmol/L, or occurrences of definite hypoglycemic symptoms. Hypoglycemia was considered severe when the event required third party assistance. Adverse events were classified as serious if they resulted in death, life-threatening experiences, hospitalization, or persistent of significant disability or incapacity.

### Statistical analysis

All data are presented as the mean and standard deviation or n and %, and were analysed using SPSS windows version 18.0. Changes in parameters from the baseline values within group were evaluated using 2-tailed paired *t*-test. Unpaired *t* test was used to compare the differences in clinical characteristics between groups at baseline and after treatment assessed for significance using for the discrete or continuous data and the chi-square test for frequency distributions. P value <0.05 was considered to be statistically significant.

## Results

### Baseline clinical characteristics

A total of 90 patients entered the trial and 84 patients (93.3%) completed the trial. Three patients dropped out the study (two changed hospital, one was lost of follow up), three patients were excluded as a result of protocol violation. Of these, 42 patients were randomly assigned to receive liraglutide to current insulin therapy (Liraglutide-added group) and 42 patients were assigned to increase the current insulin dose (Insulin-increasing group). Table 1 shows the demographic and baseline metabolic characteristics of the randomized population. At the beginning of the study, the two groups did not differ regarding anthropometric data, duration of diabetes, body weight, BMI, waist circumference, HbA1c, FBG, P2BG and total daily insulin dose. A similar proportion of patients in the two groups were using insulin glargine only (26.2% and 31.0%), NPH only (42.9% and 33.3%) and premixed insulin (31.0% and 35.7%) and type of oral antidiabetic drugs (OAD).

**Table 1:** Characteristics of the patients at baseline

	**Liraglutide added (n=42)**	**Insulin increasing (n=42)**	**P**
Age (years)	51.2 ± 10.5	52.7 ± 10.8	NS
Sex (M/F)	26/16	24/18	NS
Duration of diabetes (years)	9.1 ± 3.6	8.9± 3.6	NS
Body weight (kg)	88.6 ± 11.8	86.3 ± 10.3	NS
BMI (kg/m2)	30.4 ± 3.2	30.3 ± 3.0	NS
Waist Circumference (cm)	105.6 ± 12.6	104.6 ± 10.5	NS
Systolic blood pressure (mmHg)			
FBG (mmol/L)	8.36 ± 0.93	8.41 ± 0.86	NS
P2BG (mmol/L)	13.76 ± 1.92	13.80 ± 1.96	NS
HbA1c (%)	8.79 ± 0.86	8.69 ± 0.91	NS
Oral antidiabetic agents			
Sulfonylurea	7 (16.7%)	9 (21.4%)	NS
Thiazolidinedione	17 (40.5%)	14 (33.3%)	NS
α-glucosidase inhibitors	15 (35.7)	13 (31.0%)	NS
Glinides	16 (38.1%)	13 (31.0%)	NS
Metformin	25 (59.5%)	27 (64.3%)	NS
Insulin regimen			
Total daily insulin dose (units/day)	41.2 ± 17.4	41.6 ± 16.5	NS
Insulin glargine only	11 (26.2%)	13 (31.0%)	NS
Insulin NPH only	18 (42.9%)	14 (33.3%)	NS
Premixed insulin	13 (31.0%)	15 (35.7%)	NS

Data are expressed as mean ± S.D. or frequency [n (%)], BMI: body mass index; FBG: fasting blood glucose; P2BG: 2-hour postprandial blood glucose; HbA1c: glycosylated haemoglobin A1c; NS: not significant.

### Glycemic control and reduction of diabetes treatment

Over the 12-week treatment period, mean values of HbA_1c_, FBG and P2BG were significantly reduced in both treatment groups. There was no between-group difference in reduction in FBG and HbA_1c_. The reduction in P2BG was larger in the liraglutide-added group than in the insulin-increasing group (p=0.021) (Table 2).

**Table 2:** Changes of variables related with glucose metabolism after 12 weeks

	**Baseline (mean±s.d.)**	**12 weeks (mean±s.d.)**	**Mean changes from baseline (95% CI)**	**★Difference in mean change (95% CI)**
HbA_1c_ (%)				
Liraglutide added	8.79 ± 0.86	6.88 ± 0.64	-1.9 (-2.10, -1.72) **	-0.14 (-0.41, 0.13)
Insulin increaing	8.69 ± 0.91	6.93 ± 0.63	-1.77 (-2.00, -1.56) **	
PBG (mmol/L)				
Liraglutide added	8.36 ± 0.93	6.79 ± 0.48	-1.56 (-1.80, -1.33) **	0.13 (0.15, -0.16)
Insulin increasing	8.41 ± 0.86	6.72 ± 0.46	-1.69 (-1.90, -1.48) **	
P2BG (mmol/L)				
Liraglutide added	13.76 ± 1.92	8.39 ± 1.17	-5.40 (-5.8, -4.92) **	-0.83 (-1.59, -0.07) **#**
Insulin increasing	13.80 ± 1.96	9.26 ± 1.19	-4.54 (-5.0, -4.0) **	
Body weight (kg)				
Liraglutide added	88.6 ± 11.8	82.9 ± 11.2	-5.62 (-6.51, -4.73) **	-7.6 (-8.67, -6. 57) **##**
Insulin increasing	86.3 ± 10.3	88.3 ± 10.4	2.00 (1.52, 2.48) **	
BMI (kg/m2)				
Liraglutide added	30.4 ± 3.21	28.4 ± 3.04	-1.93 (-2.24, -1.63) **	-2.63 (-2.99, -2.67) **##**
Insulin increasing	30.3 ± 2.98	31.00 ± 2.91	0.70 (0.53, 0.86) **	
Waist Circumference (cm)				
Liraglutide added	105.6 ± 12.6	99.9 ± 11.8	-5.70 (-6.5, -5.0) **	-7.5 (-8.4, -6.6) **##**
Insulin increasing	104.6 ± 10.5	106.3 ± 10.4	1.76 (1.30, 2.23) **	

★Difference in mean change calculated as Liraglutide minus Insulin; CI, confidence interval; **p<0.01 for mean change from baseline in liraglutide and insulin; **#**p<0.05, **##** p<0.01 for the between-treatment difference.

The mean required total daily insulin dose was significantly reduced by 66% from 41.2 ± 17.4 U/day at baseline to 14.0 ± 12.5 U/day at the end of study in the liraglutide-added group (p<0.001). In contrast, the mean required total daily insulin dose was significantly increased by 28% from 41.6 ± 16.5 U/day at baseline to 53.5 ± 16.8 U/day at end of study in the insulin-increasing group (p<0.001). The mean change of total daily insulin dose was statistically significant in the two groups (p<0.001). Moreover, of the 42 patients in the liraglutide-added group, 16 (38%) came off insulin at the end of study. Furthermore, as a proportion of treatment use, add-on liraglutide treated patients had greater discontinuation of sulfonylureas (85.7% vs. 22.2%, p<0.05), Glinides (87.5% vs. 46.2%, p<0.05), α-glucosidase inhibitors (66.7% vs. 23.1%, p<0.05) but not thiazolidinediones and metformin when compared with insulin increasing treated patients.

### Body weight and waist circumference

**Figure 1:**
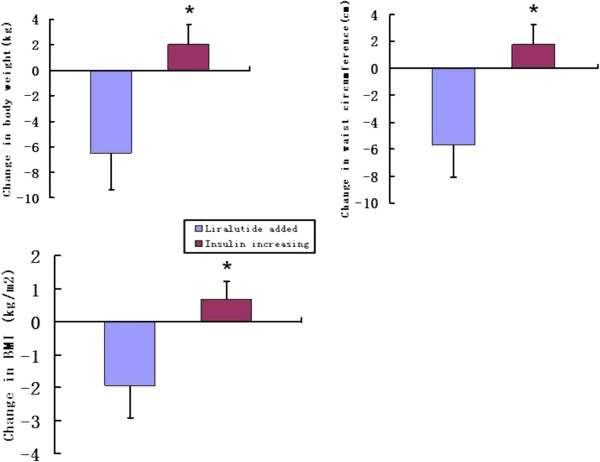
**Mean change of body weight, waist circumference and BMI in the two groups.** *p<0.001 for the between-treatment difference.

Body weight, waist circumference and BMI were significantly decreased from baseline to 12 weeks in the liraglutide-added group, the mean reductions in body weight, waist circumference and BMI were 5.62 kg, 5.70 cm and 1.93 kg/m2 respectively (Table 2). Of the 42 patients in the liraglutide-added group, 26 (62%) achieved reduction of more than 5% in body weight. In contrast, body weight, waist circumference and BMI were significantly increased from baseline to 12 weeks in the insulin increasing, the mean increases in body weight, waist circumference and BMI were 2.00 kg, 1.76 cm and 0.70 kg/m2, respectively (Table 2). The differences of mean change in body weight, waist circumference and BMI were statistically significant in the two groups (Figure 1, p<0.001).

### HbA_1c_ Target goal

**Figure 2:**
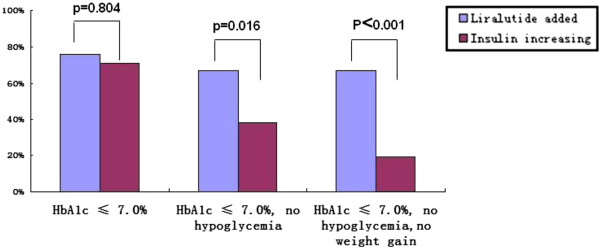
**Percentage of patients achieving composite endpoint of HbA1c ≤ 7.0%, no hypoglycemia, no weight gain** .

At the end of study, an HbA_1c_ ≤ 7.0% level was achieved by 62 (73.8%) of all the patients in the two groups. The percentage of subjects with HbA1c ≤ 7.0% with no hypoglycemia during 12 weeks treatment was significantly greater in the liraglutide-added group (67%) than in the insulin-increasing group (38%) although the percentage of subjects achieving HbA1c ≤ 7.0% in all subjects was not different between 2 groups (Figure 2). Moreover, 67% of patients in the liraglutide-added group who achieved the composite endpoint of HbA1c ≤ 7.0% with no weight gain and no hypoglycemia, this was significantly higher than 19% of patients in the insulin-increasing group.

### Hypoglycemia

No severe hypoglycemia was reported in the liraglutide-added group, while two patients in the insulin-increasing group reported severe hypoglycemia. The percentage of patients reporting minor hypoglycemic episodes during the 12-week treatment period was lower in the liraglutide-added group than in the insulin-increasing group (11.9% vs. 31.0%, p=0.033).

### Adverse events

The incidence of adverse events was higher in the liraglutide-added group than in the insulin-increasing group (57.1% vs. 33.3%, p=0.028). As expected with GLP-1 receptor agonists, gastrointestinal adverse events were most commonly reported in the liraglutide-added group, including nausea, vomiting, constipation, and diarrhea. These events were mostly mild-to-moderate in severity and typically resolved after the first 4–8 weeks. There were no patients withdrawn from the 12 week study as a result of medication related adverse events in the two groups.

## Discussion

Compared with increasing the insulin dose therapy, the present study demonstrated the beneficial effects of adding the long-acting GLP-1 analog liraglutide to established insulin therapy, which resulted in a significant improvement in glycemic control, reduction in insulin requirement, lower incidence of hypoglycemia events and weight loss in the Chinese patients with poorly controlled T2D and abdominal obesity. Although beneficial effects of combination use of GLP-1 receptor agonists (liraglutide or exenatide) and insulin were observed in previous small studies [17-19], most of these are retrospective studies. This study is the first designed study using the insulin dose-increasing approach as an active comparator.

The data from all six LEAD trials have been analysed in pooled meta-analysis which has shown that addition of liraglutide to existing OAD therapy resulted in mean HbA1c reductions of approximately 1.4% and mean HbA1c level of 7.1%, and the proportion of subjects with HbA1c <7% was 59% at the end of trial [20]. In the present study, addition of liraglutide to the established insulin therapy resulted in a significant 1.9% decrease in HbA1c and mean HbA1c level of 6.88%, and 76.2% of patients treated with addition liraglutide achieved an HbA_1c_ ≤ 7.0% level at the end of study. Patients in the insulin-increasing group experienced similar glucose control compared with that in the liraglutide-added group, but when compared with the composite endpoint including HbA1c ≤ 7.0% with no weight gain and no hypoglycemia, there is a significant difference between two groups. The composite endpoint goal was achieved by 67% of patients in the liraglutide-added group which was significantly higher than that 19% of patients in the insulin-increasing group. In this study, we also found that liraglutide-added treatment improved postprandial glucose control better than insulin-uptitration. One possible explanation for this beneficial effect is that liraglutide can suppress glucagon secretion in addition to increasing the physiological insulin secretion [7,8]. Furthermore, add-on liraglutide treated patients had lower rate of hypoglycemic events and greater insulin and OAD discontinuation than did patients treated with increased insulin doses. This suggests that the addition of liraglutide to insulin therapy might be a good practice to attain glucose control.

In the Diabetes Control and Complications Trial, subjects assigned to intensive therapy experienced a three-fold increased risk of severe hypoglycemic events [21]. A similar finding was also obtained in the UK Prospective Diabetes Study [22]. In the present study, no severe hypoglycemia events were reported in liraglutide-added group, while two patients in the insulin-increasing group reported severe hypoglycemia. In addition, the percentage of patients reporting minor hypoglycemic episodes during the 12-week treatment period was significantly lower in the liraglutide-added group than in the insulin-increasing group. The finding is consistent with the previous studies [3,10-13] and highlights a second advantage to the liraglutide adding strategy. The lower risk of hypoglycemia with liraglutide administration may be explained by its stimulation of insulin release and glucagon suppression in a glucose-dependent manner [23]. Obese patients with T2D are at high risk for CVD [4]. Given that recent studies have shown an association between hypoglycemic events and risk cardiovascular events [24], our study may suggest that adding liraglutide to insulin is advantageous from a cardiovascular standpoint owing to its reduced frequency of hypoglycemia compared to treatment with insulin only. In addition to a lower risk of hypoglycemia, liraglutide may favorably affect several CV risk factors, such as blood pressure, lipid profiles, and body weight [11-17,25]. A recent meta-analysis of cardiovascular safety by exenatide showed that patients treated with exenatide twice daily were less likely to have a CVD event than were treated with other glucose-lowering therapies [26]. The direct cardiovascular benefits of adding liraglutide should be verified in prospective clinical trials.

Insulin, the most effective therapeutic agent for lowering the blood glucose, is particularly associated with weight gain [27,28] and especially causes undesirable weight gain in an already obese population. Therefore it increases physicians’ reluctance to intensify treatment and decreases patient adherence. All OADs for the treatment of T2D are associated with either weight gain or weight neutrality, except for GLP-1 receptor agonist class which resulted in weight loss through central appetite suppression, leading to reduced energy intake. Therefore, the third advantage to the liraglutide adding strategy was the reduction of body weight. In the present study, a mean weight reduction of 5.62 kg was observed in the liraglutide-added group at the end of the study. It is greater than that observed in any of the preclinical trials of liraglutide as monotherapy or as add-on therapy to oral agents [10-13] and the trial performed in Asian countries [3,29]. As seen in a recent observational study which combined liraglutide and insulin therapy, the weight loss seen in our study may be explained by a combination of reduced caloric intake caused by the appetite-suppressing effect of liraglutide coupled to reduced lipogenesis achieved by insulin reduction [18]. Moreover, the mean waist circumference reduction of 5.7 cm was most impressive in the liraglutide-added group. Visceral fat in particular increases the risk of CVD, with a recent meta-regresion analysis reporting a 2% increase in the relative of a CVD event for 1-cm increase in waist circumference [6]. Therefore, mean 5.7 cm reduction of waist circumference in the liraglutide-added group was sufficient to reduce the risk of cardiovascular events.

Participants who are T2D and abdominal obesity are likely to develop hypertension, hypercholesterolaemia, liver disease and eventually CVD. Accordingly these participants have a considerably elevated risk of morbidity and mortality. Unfortunately, traditional treatments for these patients are associated with weight gain and hypoglycemia that limit the number of patients reaching acceptable therapeutic goals. The present study provides evidence that liraglutide, when given to insulin-treated obese patients with T2D, results in clinically relevant beneficial effects on body weight and waist circumference reduction, lower risk of hypoglycemia and insulin dose reduction in addition to improved glycemic control.

## Conclusions

In conclusion, adding liraglutide to insulin therapy provides a better chance of achieving good glycemic control with a lower daily insulin dose and fewer hypoglycemic events compared to increasing insulin dose. This regimen also resulted in a significant reduction in body weight and waist circumference, suggesting that the addition liraglutide to insulin treatment in obese patients with T2D maybe a more effective option than increasing the insulin dose alone. Larger randomized, prospective studies are required to define the best practices for combination therapy with insulin and liraglutide and to evaluate its long-term cardiovascular effects.

## Abbreviations

AEs: Adverse events; ALT: Alanine aminotransferase; AST: Aspartate aminotransferase; BMI: Body mass index; CVD: Cardiovascular disease; FBG: Fasting blood glucose; GLP-1: Glucagon-like peptide-1; HbA1c: Glycosylated hemoglobin; LEAD: Liraglutide Effect and Action in Diabetes; OAD: Oral antidiabetic drugs; P2BG: 2 hour postprandial blood glucose; T2D: Type 2 diabetes.

## Competing interests

The authors declare that they have no conflicts of interest.

## Authors’ contributions

Li CJ and Yu P acquired and analyzed data, and wrote the manuscript. Yu DM conceived study, analyzed data and reviewed the manuscript. Li J, Zhang QM, Lv L, Chen R and Lv CF acquired and researched data. All authors read and approved the final manuscript.
